# Enhanced Humidity Sensing Performance of Fe_2_O_3_:ZnO/MWCNTs Nanocomposite: Fabrication and Characterization

**DOI:** 10.1021/acsomega.6c02389

**Published:** 2026-05-19

**Authors:** Artak Sayunts, Gevorg Shahkhatuni, Rima Papovyan, Zarine Simonyan, Davit Kananov, Gohar Shahnazaryan, Gabriel Gevorgyan, Andranik Grigoryan, Dušan Kopecký, Mikayel Aleksanyan

**Affiliations:** † Center of Materials Science and Nanotechnologies, 105430 Yerevan State University, 1 Alex Manoogian, 0025 Yerevan, Armenia; ‡ Department of Mathematics, Informatics and Cybernetics, Faculty of Chemical Engineering, 52735 University of Chemistry and Technology Prague, Technická 5, 166 28 Prague 6 Prague, Czech Republic

## Abstract

This work was dedicated to the synthesis and characterization of Fe_2_O_3_:ZnO/MWCNTs (multiwalled carbon nanotubes) nanocomposite as a high-performance humidity sensor. The Fe_2_O_3_:ZnO composite was prepared by a simple and inexpensive solid-phase synthesis method by using thermal annealing. The sensor fabrication involved the deposition of the Fe_2_O_3_:ZnO thin film and MWCNTs onto the sensor substrates using radio frequency (RF) magnetron and e-beam deposition methods, respectively. Various characterization techniques, including scanning electron microscopy, profilometry, energy-dispersive X-ray spectroscopy (EDX), transmission electron microscopy, and X-ray photoelectron spectroscopy, were employed to investigate the material properties of the Fe_2_O_3_:ZnO/MWCNTs nanocomposites. The sensor responses were measured toward a relative humidity range of 2.2%–98%, where their values changed from 1.5 to 5, respectively. The high selectivity and temporal stability of the sensor were confirmed by the experimental results. Besides, the Nyquist curves of the Fe_2_O_3_:ZnO/MWCNTs material were extracted using the impedance investigation, and an equivalent electrical circuit was proposed. Thus, this work will contribute to the implementation of highly efficient and viable sensors in humidity detection systems.

## Graphical abstract

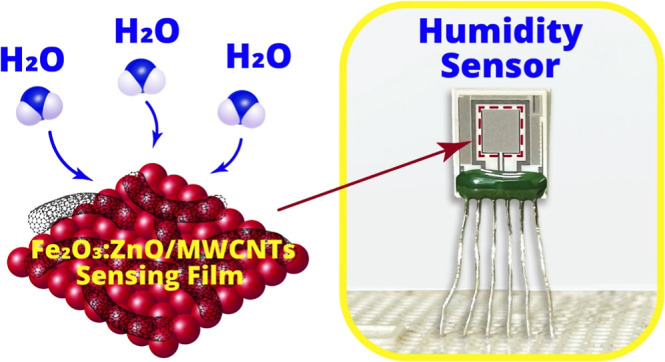

## Introduction

Monitoring relative air humidity and measuring low water vapor concentrations in indoor and outdoor spaces of human activity are considered noteworthy challenges.^1,2^ The need for accurate and selective sensors that detect water vapor is evident in biomedical diagnostics, material preservation, agricultural management, and climate control.^3^ Humidity control is also important in hydroelectric and thermal power plants, industrial processes, mining, food processing, and beverage production.^4,5^ It also plays a crucial role in the operation of hydrocarbon-fueled vehicles, where monitoring the moisture content in the engine exhaust gas feedback regulates fuel consumption.^6^ Thus, high-performance humidity sensors are in demand in almost all areas of human activity (Figure 1), which forces the introduction of new advanced technologies and materials to produce sensitive structures that meet current requirements.

**Figure 1:**
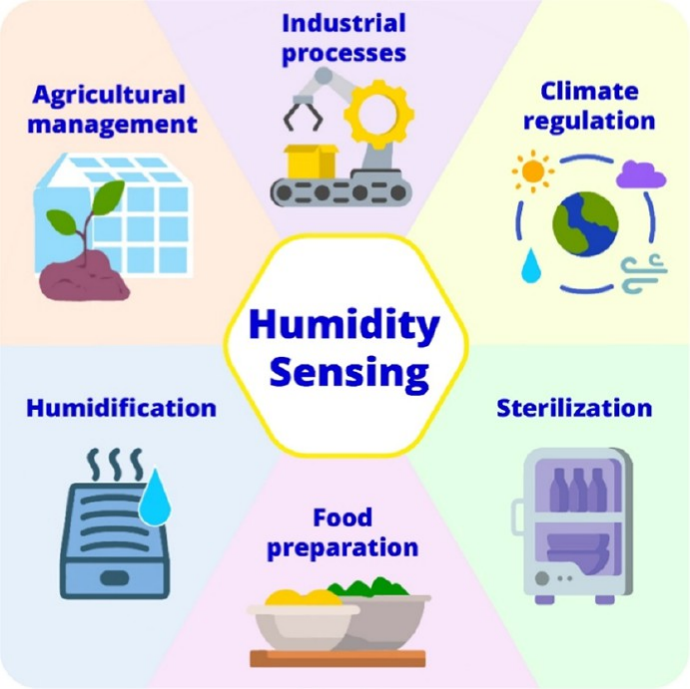
Application fields of humidity sensors.

Humidity sensors are classified as capacitive, optical, electrochemical, voltage-based, impedance-based, colorimetric, and resistive types, each of which converts the presence of humidity into an electrical signal by changing some physical parameter.^7–9^ Due to their small size, advanced fabrication technology, and adaptability to micro- and nanotechnologies, resistive sensors are characterized by high sensitivity, stability, performance, chemical activity, and low cost.^10,11^ Currently, various approaches exist for the design and fabrication of resistive sensors; however, the key challenge lies in controlling the material properties and morphologies of the sensitive nanostructures.^12–14^ The sensitive materials used here are classified into these main groups, such as perovskite oxides, metal oxides, polymeric compounds, and carbon-based materials, the applicability of which is conditional depending on the intended use of the device.^15,16^ As materials with high temporal stability, low cost, chemically and mechanically stable, environmentally friendly, and highly sensitive, metal oxides are increasingly used as preferred materials in humidity sensors.^17^ Despite the advanced properties of metal oxides, their undoped states react rather weakly with the gaseous phase and have a rather high electrical resistance that is difficult to measure. Due to these issues, doped metal oxides and composite materials have been synthesized as gas-sensitive structures in the past decade.^18^ Metal oxide nanostructures with advanced gas-sensing properties, such as Fe_2_O_3_ and ZnO, are widely used here.^19,20^ Fe_2_O_3_ has acceptable advantages for application in gas sensors, such as high chemical activity toward reducing gases, easy control of parameters by introducing dopants, compatibility with modern micro/nanosystems, and low-cost synthesis (band gap: 2.43–3 eV, melting point: 1475 °C-1565 °C, density: 5.242 g/cm^3^, and refractive index: 1.45–3.23).^21–23^ The introduction of zinc oxide into this basic gas-sensitive matrix leads to an increase in sensitivity and selectivity, since ZnO itself is distinguished by its wide bandgap energy with excellent oxidation–reduction properties (band gap: 3.37 eV, transmittance: 80%–90%, exciton binding energy: 60 meV, melting point: 1975 °C, density: 5.605 g/cm^3^, crystal structure: *a* = 3.25 A°, *c* = 5.2A°, and mechanical hardness: 4.5 on the Mohs scale),^24–27^ chemical and mechanical strength, high sensitivity to reducing gases, environmental friendliness, and low cost.^28–32^

To obtain improved gas-sensing results, it is also necessary to refine the morphology of the sensitive matrix, leading to an increase in the effective surface area. This leads to an increase in the gas absorption capacity of the sensitive layer, accompanied by more intense chemical reactions and diffusion processes.^33^ Tuning the morphology of a gas-sensitive film can be most effectively achieved using one-dimensional (1D) and two-dimensional (2D) systems, for example, by introducing carbon-based nanostructures into the main metal oxide nanostructure.^34^ In particular, carbon nanotubes are well suited for this purpose due to their large effective surface area, high flexibility, mechanical robustness, and long-term stability. Besides, due to the high charge carrier mobility of CNTs, their introduction into basic metal oxides also leads to an increase in system performance.^35,36^

Advanced humidity-sensing structures based on various nanocomposites have been developed and extensively studied; however, they have not yet fully satisfied the current performance requirements. Eom et al.^37^ fabricated a chemoresistive humidity sensor based on ultrathin V_2_O_5_·nH_2_O nanobelts (NBs) using the liquid-phase exfoliation method. The sensor response to 10% relative humidity (RH) was about 24% with the response and recovery times of 21 and 356 s, respectively. Han et al.^38^ synthesized a ZnS/Ti_3_C_2_T_*x*_ humidity-sensitive material by a hydrothermal method. The sensor exhibited a 14% response to 54% RH, while the low detection limit was 10.9%. Manjunatha et al.^39^ synthesized a Mg_1–*y*_Li_*y*_Fe_2_O_4_ (*y* = 0, 0.01, 0.03, and 0.05) material with humidity-sensing properties using the solution combustion synthesis route method. It had a humidity sensing range of 11%–97% with a maximal sensing response of 93%. Thus, sensors reported in the literature exhibited a range of limitations, including insufficient response enhancement, relatively high minimum detection limits, and prolonged response and recovery times, highlighting the challenges that remain unresolved in the field.

In this study, Fe_2_O_3_:ZnO/MWCNTs nanocomposite material was fabricated by RF magnetron and e-beam deposition methods as a humidity-sensitive material. The sensing material was subjected to scanning electron microscopy (SEM), transmission electron microscopy (TEM), EDX, and X-ray photoelectron spectroscopy (XPS). The sensor demonstrated pronounced sensitivity and selectivity across a broad range of water vapor concentrations (2.2%–98% RH). In this system, the metal oxide composite (Fe_2_O_3_:ZnO) acts as the gas-to-electrical signal transduction matrix, while MWCNTs enhance the effective surface area, thereby increasing the gas adsorption capacity. The novelty of this work lies in the successful integration of magnetron-sputtered Fe_2_O_3_:ZnO nanostructured films with MWCNT networks combined with UV-assisted operation, enabling the development of a highly homogeneous nanocomposite humidity sensor exhibiting enhanced sensitivity, wide-range linear detection, low hysteresis, rapid recovery, and excellent stability while simultaneously providing an equivalent electrical model and comprehensive insight into the humidity sensing mechanism.

## Experimental Section

### Materials

Fe_2_O_3_ (15–45 nm, 99.9% purity) and ZnO (15–45 nm, 99.9% purity) nanopowders were purchased from Alfa Aesar (Haverhill, MA, USA) for RF target fabrication. The MWCNTs were obtained from Nanochemazone Inc. (Canada, Alberta) to enhance the sensor’s active surface. The palladium target (Pd, 99.95% purity) was acquired from Goodfellow Ltd. (Shanghai, China) for sensor surface sensitization. Factory-designed sensor substrates were purchased from TESLA BLATNÁ (Blatná, Czech Republic). The overall dimensions of the alumina substrate were 6.2 × 5.2 mm^2^, while the active layer sides were 2.4 × 2 mm^2^ (electrode and gape sizes were 15 μm.).

### Fabrication of the Fe_2_O_3_:ZnO Material

A presynthesized Fe_2_O_3_:ZnO composite material was used as the target for RF magnetron sputtering. The target was prepared by a simple and inexpensive solid-phase synthesis method using thermal annealing. Appropriate amounts of Fe_2_O_3_ and ZnO (6 at. %) nanopowders were thoroughly mixed and mechanically milled for more than 20 h until they became a homogeneous mixture. The powdery mass was then pressed into a cylindrical tablet with a diameter of 50 mm and a thickness of 4 mm, using the dimensionally adjusted magnetron head of the RF sputtering. It underwent preliminary annealing to a temperature of 800 °C, which lasted 10 h. Then, the resulting tablet was subjected to high-temperature (up to 1250 °C) and long-term (24 h) annealing using a software-controlled furnace (Nabertherm, HT 04/16). All thermal annealing regimes are listed in Table 1. At higher temperatures, the two materials interacted at the atomic level to form a polycrystalline ceramic solid suitable for use as a vacuum sputtering target. The material preparation phase was completed by subjecting the Fe_2_O_3_:ZnO tablet to mechanical and chemical cleaning. The chemical cleaning was performed by exposing it to ultrasound for 20 min, then boiling in alcohol for 1 h and finishing by leaving the sample in boiling toluene for 30 min.

**Table 1:** Thermal Annealing Regimes for Synthesizing of Fe_2_O_3_:ZnO Material

regime [N]	annealing temperature [°C]	annealing duration [h]
1	25–800	10
2	800–900	12
3	900–1000	14
4	1000–1150	18
5	1150–1250	24
6	1250–25	10

### Fabrication of the Fe_2_O_3_:ZnO/MWCNTs Sensor

The sensor manufacturing process began with chemical cleaning of the sensor substrate’s active surface using alcohol and toluene solutions. The substrate surface was then subjected to dry ion cleaning before the main deposition process was carried out. In the sputtering (vacuum) chamber, the RF and DC heads were equipped with the Fe_2_O_3_:ZnO and Pt targets, respectively. In the case of a generator producing an alternating field of 70 W, the ignited argon plasma was directed at the Fe_2_O_3_:ZnO target, detaching nanoparticles from it. The nanoparticles, transported through a vacuum environment, condense onto the sensor substrate, forming a dense film during a deposition period of 25 min. Then, in the same vacuum environment, the plasma was directed to the Pt target and, as a result of sputtering for 5 s, catalytic nanoparticles were deposited on the surface of the Fe_2_O_3_:ZnO film. The effective surface area of the gas-sensitive film was artificially increased by depositing MWCNTs, thereby using the e-beam deposition method. The deposition duration was 3 min, and to address adhesion issues, the substrate temperature was maintained at 250 °C throughout the deposition process. In the final stage of production, the sensor was subjected to thermal annealing at 300 °C for 4 h, which allowed for stabilization of the sensor parameters. The entire sensor manufacturing process is depicted in detail in Figure 2, and the sensor’s structural block diagram is shown in Figure 3.

**Figure 2:**
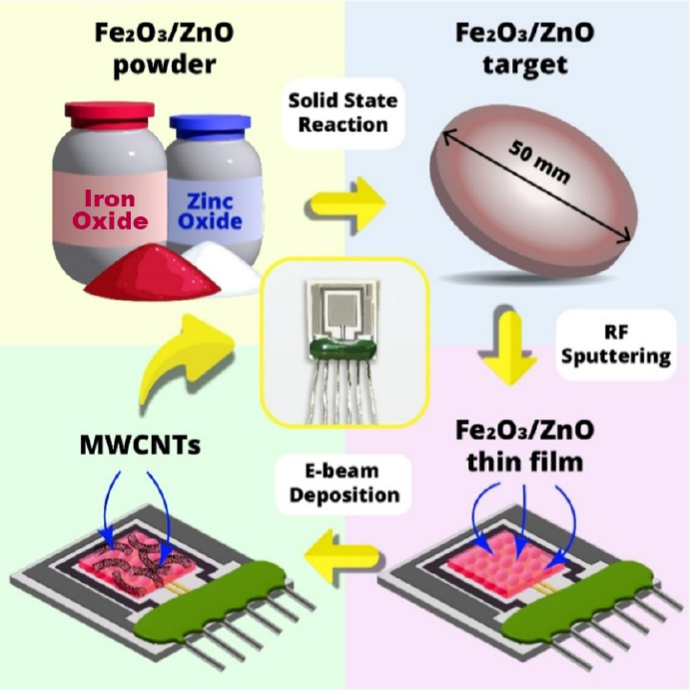
Fabrication steps and the actual photograph of the Fe_2_O_3_:ZnO/MWCNTs sensor.

**Figure 3:**
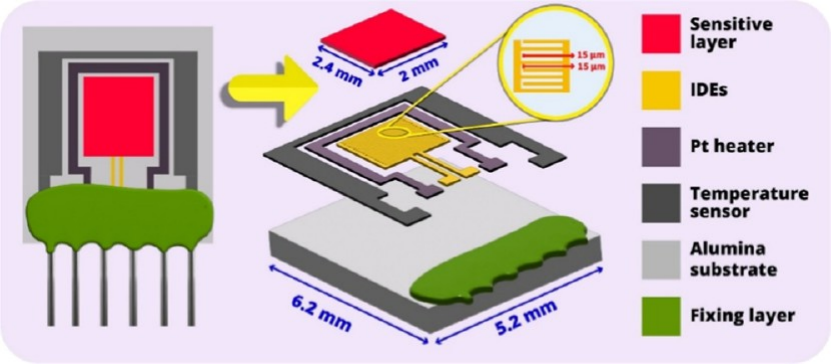
Schematic illustration of the humidity sensor.

### Humidity Measurement Setup

A laboratory-made gas testing system (Figure 4) was used to measure the humidity response of the Fe_2_O_3_:ZnO/MWCNTs sensor. Electrical connections between the six sensor pins (heater, temperature sensor, and resistance measuring electrodes) and the power supply (Twintex TP-2303) were established, and the measured data collection was made using a digital multimeter (Keithley DMM 7510). To illuminate the sensor surface with UV rays, a UV LED (λ = 395 nm) was placed 1.5 cm away from the active surface of the sensor capable of illuminating with an intensity of 2.5 mW/cm^2^. The humidity level in the chamber at different concentrations was provided by evaporating pure water, the number of moles of which was previously calculated. The sensor response was considered the ratio of the sensor resistances in air and in the presence of water vapor, typical for the resistive sensors.^40^

**Figure 4:**
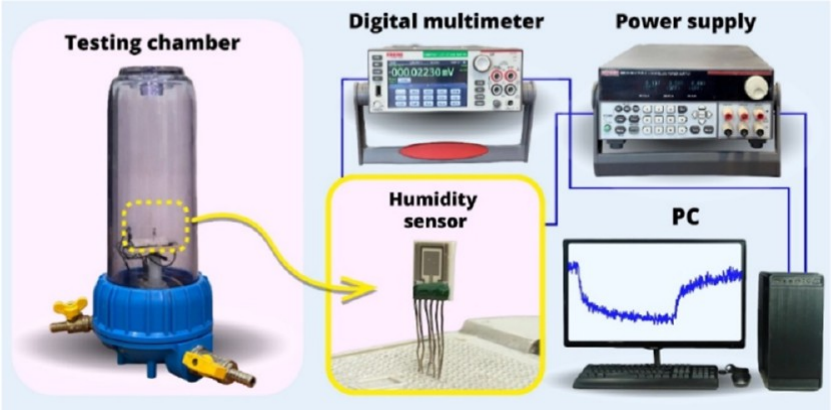
Schematic block diagram of the humidity testing system.

## Results and Discussion

### Material Characterization

The Fe_2_O_3_:ZnO nanopowders were subjected to structural analysis to reveal the sizes of the nanoparticles presented here. The grain sizes in the nanopowder mainly ranged from around 100–120 nm (Figure 5), which was quite suitable for subsequent thermal annealing to form a polycrystalline target. Against the background of black spherical nanoparticles, which can be attributed to iron oxide, zinc particles with an almost hexagonal structure were visible, contrasting with white. During the process of mechanical grinding and mixing, moisture was absorbed from the atmosphere into the nanopowder, which affected the subsequent solid-phase reaction synthesis process.^41^ With larger particle sizes (>100 nm), that is, greater porosity, the ability to absorb more moisture increases, which can subsequently lead to the formation of cracks in the solid target during the solid-phase synthesis process.

**Figure 5:**
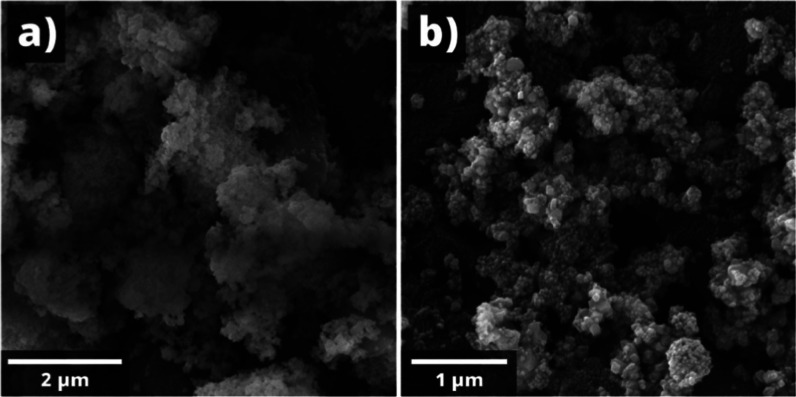
SEM images of the Fe_2_O_3_:ZnO powder with the scale bars of 2 μm (a) and 1 μm (b).

One of the main advantages of RF magnetron sputtering is the homogeneous deposition of nanoparticles even from a high-resistance target.^42^ Homogeneous deposition of the film is enabled by homogeneous plasma and magnetic fields; consequently, nanoparticles detached from the target underwent uniform condensation on the substrate. The Fe_2_O_3_:ZnO target, having sufficient compactness and strength, withstood the magnetron sputtering process fully, ensuring a stable flow of nanoparticles to the substrate. The substrate temperature, which was higher than room temperature (250 °C), contributed to the good adhesion of the film and energy efficiency of its formation. Thus, the Fe_2_O_3_:ZnO film was sufficiently homogeneous, as reflected in its SEM images (Figure 6a, b).

**Figure 6:**
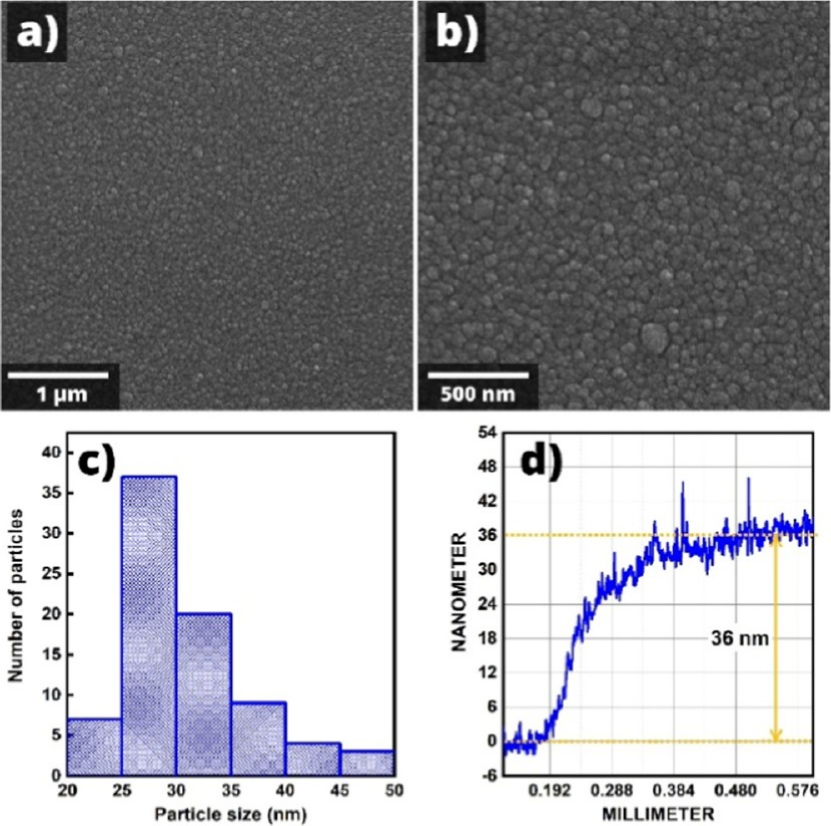
SEM images (a, b), particle size histogram (c), and thickness measurement result (d) of the Fe_2_O_3_/ZnO film.

The sizes of the film grains are of crucial importance in the sensing mechanism, as they determine the gas sensitivity and temporal stability of the sensor.^43^ The histogram distribution of grain sizes in the film allowed us to estimate their diameter as 25–28 nm (Figure 6c), fully validating the *D* < 2*L*_*D*_ condition.^44^ This was definitely a favorable condition for obtaining high sensitivity, considering the Debye length of the Fe_2_O_3_:ZnO system at 250 °C (*L*_*D*_∼15–20 nm).^45^ Besides, sensitivity and stability can also be greatly influenced by tanning the thickness of the sensitive film.^46^ In the magnetron sputtering technique, with a constant generator power, the film thickness is mainly controlled by varying the deposition time. In our case, a 25 min sputtering time resulted in a film with a thickness of about 36 nm (Figure 6d), which demonstrated sufficient temporal stability.

To evaluate and improve the gas-sensing characteristics of the sensor, it is crucial to determine the crystal structure of the sensitive material. For this purpose, the Fe_2_O_3_:ZnO material was thoroughly examined by the TEM technique. The hexagonal traces of ZnO, whose sizes were about 30–40 nm, were clearly visible in the low-resolution TEM images (Figure 7a, b). The interplanar distances for Fe_2_O_3_ and ZnO material were visible in a high-resolution TEM (HRTEM) image, attributed to 0.5 and 0.25 nm,^47^ respectively (Figure 7c). The polycrystalline nature of the Fe_2_O_3_:ZnO material was confirmed^48^ by the selected area electron diffraction (SAED) pattern (Figure 7d).

**Figure 7:**
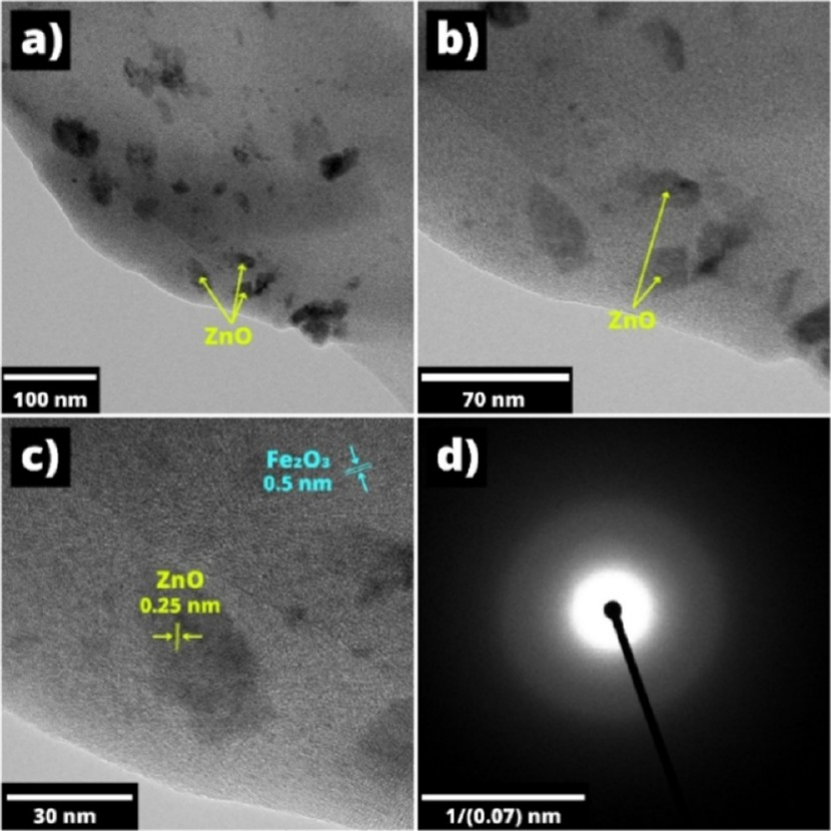
Low (a, b) and high-resolution (c) TEM images and SAED pattern (d) for the Fe_2_O_3_:ZnO material.

The deposition of functionalized MWCNT islands in the main gas-sensing matrix was quite effective in terms of improving the sensor’s sensitivity, stability, and speed.^49^ The MWCNTs used in sensitive materials as an important component in the production of composite materials have unique morphological characteristics. They are a collection of nanotubes of different diameters in the form of a honeycomb-like structure (Figure 8), which can undergo morphological transformations as a result of technological processes. Thus, SEM images of the chemical vapor deposition–grown nanotubes revealed that they were relatively long, with lengths of 15–30 μm and diameters in the range of 50–200 nm.

**Figure 8:**
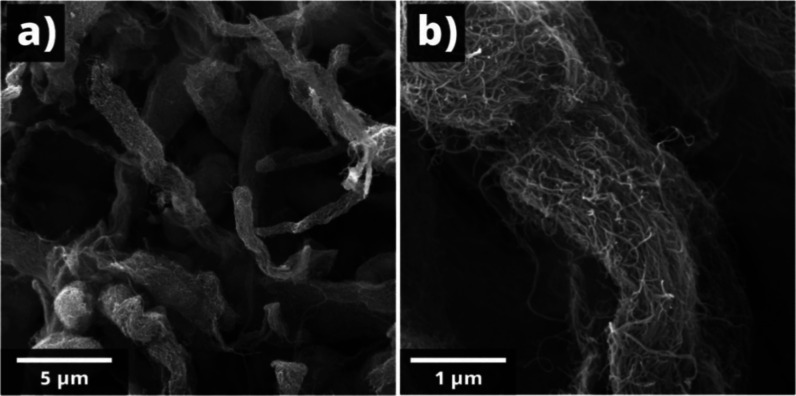
SEM images for MWCNTs with the scalebar of 5 μm (a) and 1 μm (b).

The pronounced dimensions of the MWCNTs are definitely favorable in the gas-sensing mechanism, in terms of the adsorption of gas molecules and their diffusion into the inner regions of the nanotubes. Besides, these results were also in good agreement with the TEM measurements of the MWCNTs. The nanotubes were cut into pieces, and their morphology was revealed in low- and high-resolution TEM images (Figure 9). Crystalline lattice disruptions and more polycrystalline structures were observed on the outer walls of the nanotubes. This was presumably due to the characteristics of nanotube growth and the dynamic deviation of the longitudinal dimensions during the growth process.^50^ To obtain a larger effective surface area and greater adsorption capacity of gas molecules, it is more appropriate to use such unregulated systems.^51^

**Figure 9:**
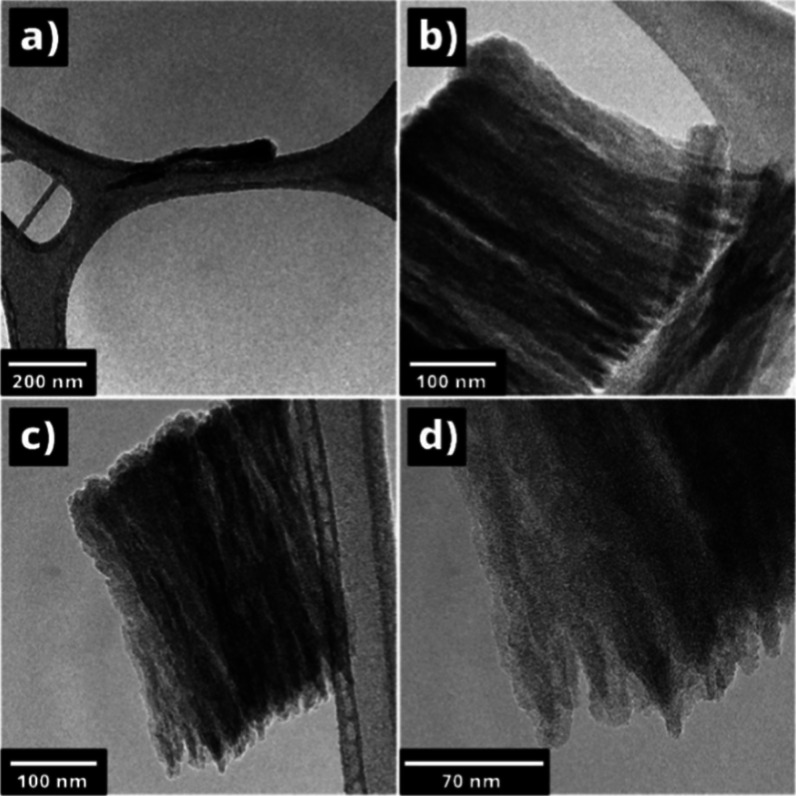
Low (a, b, c) and high (d) resolution TEM images for the MWCNTs.

The EDX spectrum of the Fe_2_O_3_:ZnO/MWCNTs structure was also carefully examined, revealing the elements present here (Figure 10). Here, peaks reflecting iron (Fe), zinc (Zn), oxygen (O), and carbon (C) were present. The relative weight percentages of the elements were also measured here, with iron accounting for the largest proportion (59.85 wt %). The second most abundant compound was expressed for oxygen (25.55 wt %), which was due to the oxygen composition contained in oxides and oxygen species adsorbed from the environment. Besides, due to ZnO and MWCNTs, here the Zn and C contents were 12.28 and 2.32 wt %, respectively.

**Figure 10:**
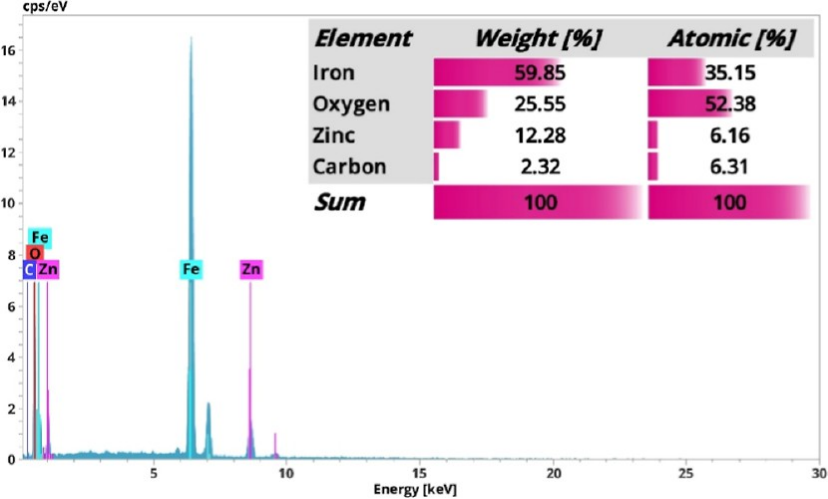
EDX spectrum of Fe_2_O_3_:ZnO/MWCNTs material.

The homogeneous distribution of individual elements in the base material is a crucial attribute in terms of both gas-sensing parameters and material stability. Thus, the distribution characteristics of the elements were assessed by using the EDX elemental mapping images. The obvious homogeneous distribution of constituent elements available in the Fe_2_O_3_:ZnO/MWCNTs material is well expressed in Figure 11.

**Figure 11:**
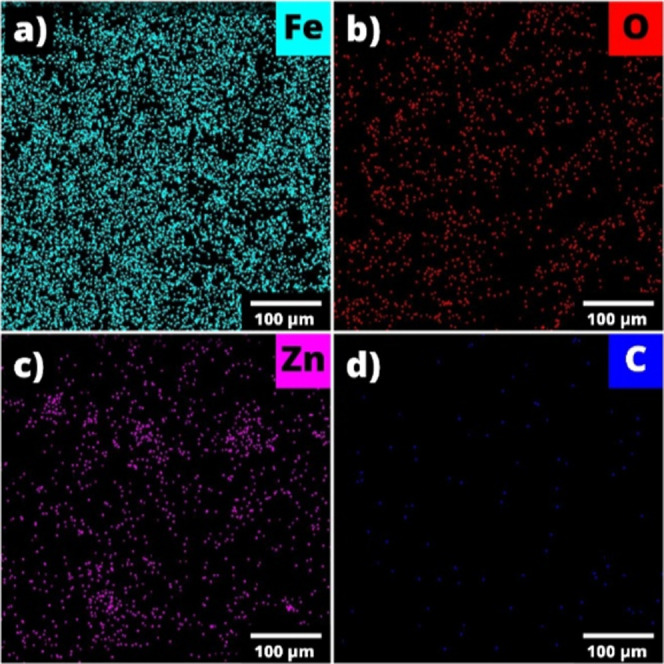
EDX elemental mapping analysis of Fe (a), O (b), Zn (c), and C (d) in the Fe_2_O_3_:ZnO/MWCNTs material.

The XPS survey spectrum of the Fe_2_O_3_:ZnO/MWCNTs material is presented in Figure 12a, representing the Fe, Zn, O, and C elements. The electron binding energies of Fe in its high-resolution spectrum were 710.5 and 724.3 eV corresponding to 2p3/2 and 2p1/2, respectively (Figure 12b). This coincided with the typical nature of γ-Fe_2_O_3_.^52^ The high-resolution spectrum of Zn represented to 1021.8 eV (Zn 2p3/2) and 1045.0 eV (2p1/2) peaks (Figure 12c) revealed ZnO material.^53^ There were also two peaks in the high resolution O 1s spectrum at 530.5 and 532 eV (Figure 12d) attributed to oxygen in Fe_2_O_3_ and ZnO, respectively.^54^ The pronounced peak of C 1s was registered at 285.3 eV (Figure 12e) as a C–C bond attributed to MWCNTs.^55^

**Figure 12:**
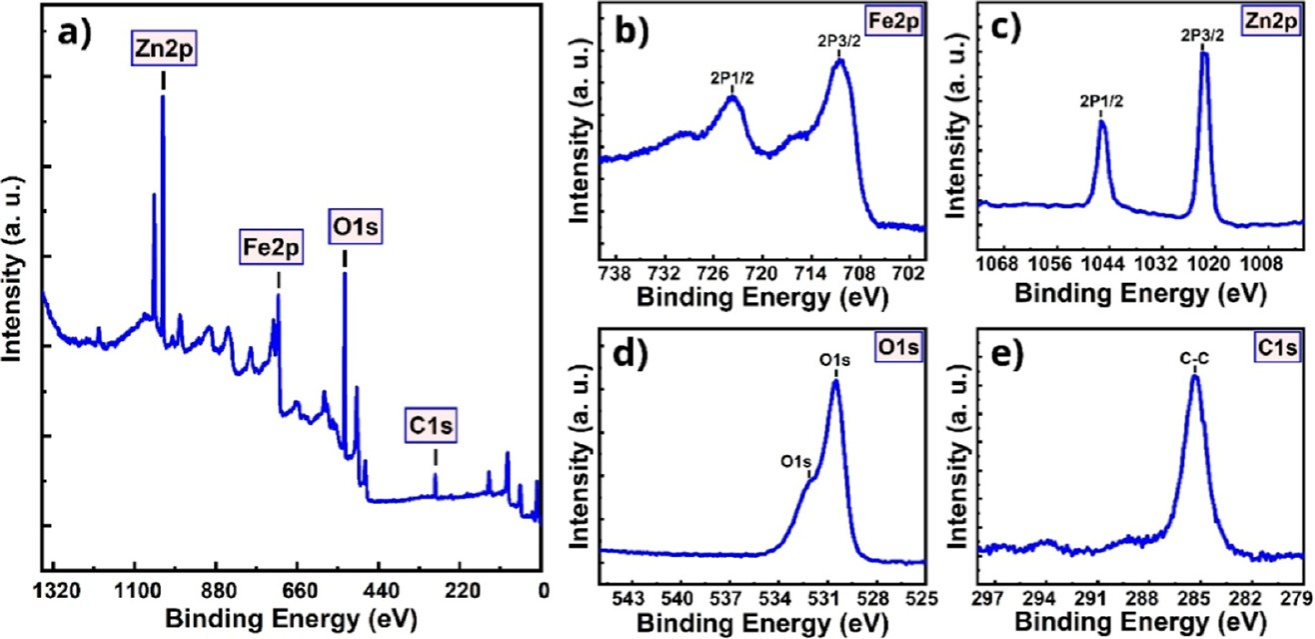
XPS survey spectrum (a), high-resolution XPS spectrum of Fe 2p (b), Zn 2p (c), O 1s (d), and C 1s (e) for the Fe_2_O_3_:ZnO/MWCNTs material.

### Humidity Sensing Characteristics

The gas-sensing characteristics of the Fe_2_O_3_:ZnO/MWCNTs sensor were investigated under various conditions by using both thermal heating and heating combined with UV irradiation. At room temperature, the sensor showed a fairly fast and large response to UV irradiation, which is a common behavior for nanostructures with semiconductor properties (Figure 13a). As the temperature increased, the UV response decreased because at high temperatures, the thermal energy already generated a sufficient number of free carriers (electrons/holes), while the light-activated charge carriers were no longer capable of making a significant quantitative change in the overall conductance. At 250 °C, the UV response was almost completely extinguished without changing the sensor resistance. Despite this, the sensor’s response to water vapor under these conditions was significantly better than without UV, where the sensor response was extremely poor. To determine the sensor response characteristics at different temperatures, the sensor response values were measured over a temperature sweep from 25 to 300 °C. The sensor showed sensitivity even at room temperature under the influence of UV irradiation, despite being extremely slow. As the temperature rose, the sensor response increased, reaching a peak at 250 °C (Figure 13b). This is the turning point at which the adsorption and chemical reactions of the target gas on the gas-sensing surface occur with maximum efficiency.^56^ At higher temperatures (>250 °C), a significant portion of water vapor cannot adsorb to the surface, leading to fewer molecules in the chemical reaction processes. Thus, the most preferred point on the temperature/response curve for humidity recording and concentration measurement was 250 °C, which was considered the operating temperature. The dynamic change in sensor resistance at 250 °C in the presence of 22% RH is shown in Figure 13c. It was observed that the resistance change induced by water vapor reached a steady-state value at 250 °C, whereas in the absence of the target gas, the sensor exhibited negligible recovery toward its baseline resistance. Due to the lack of natural recovery of the sensor, the pulsed heating method was used. At the moment of removing the gas effect, the sensitive film of the sensor was instantly heated up to 300 °C for a few seconds, as a result of which intensive desorption of water molecules from the surface occurred. However, in parallel with this, free charge carriers were generated in the semiconducting layer at a higher temperature, which significantly reduced the resistance of the sensor. This was expressed in the form of a beak-shaped peak presented in the dynamic resistance curve (Figure 13c). After pulsed heating was stopped, the sensor resistance quickly recovered to its initial baseline value. In all other dynamic resistance curves presented in this paper, these artificial thermally generated peaks were removed by approximating them with their corresponding recovery times.

**Figure 13:**
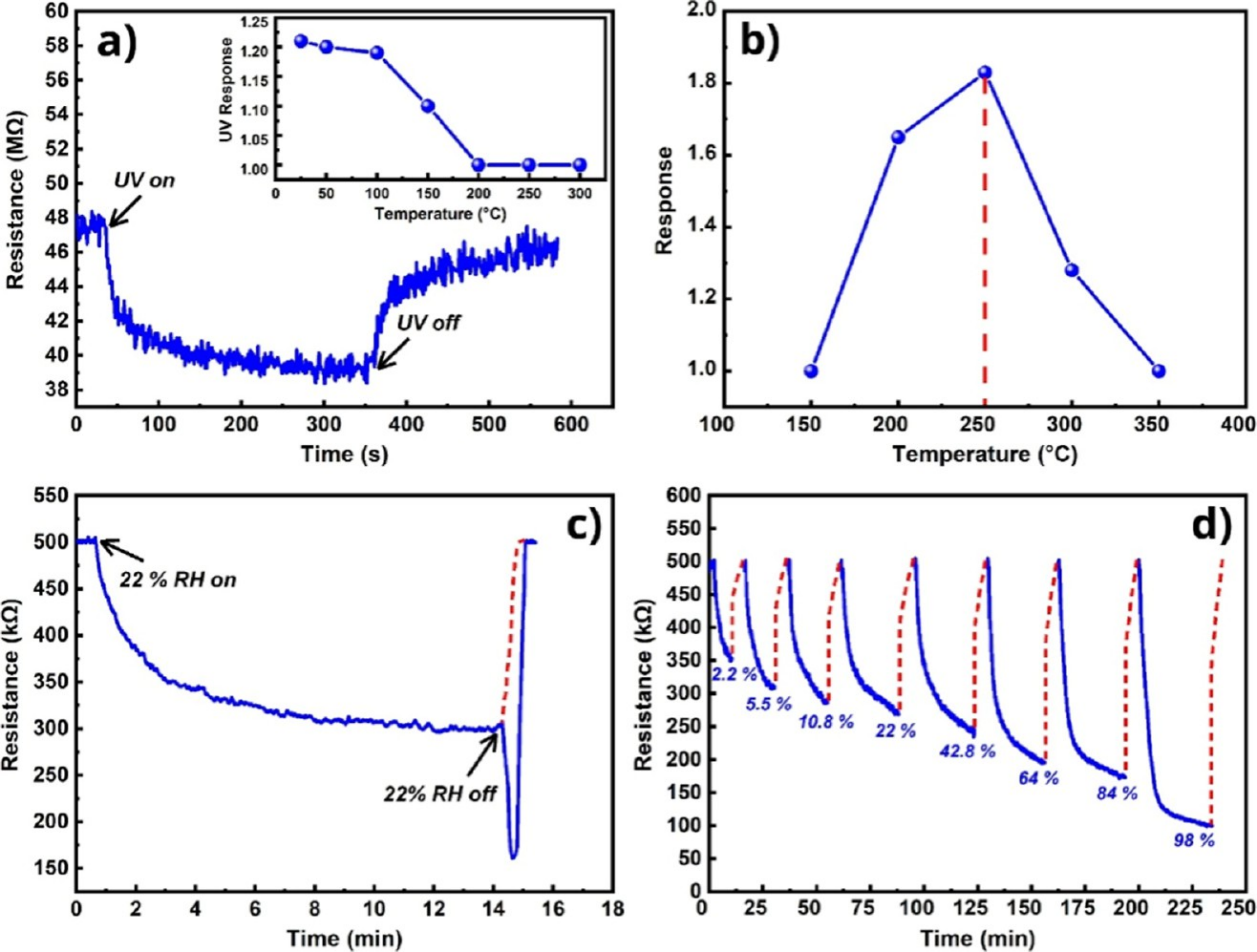
(a) UV response at room temperature (the inset is UV response at different temperatures), (b) sensor response vs operating temperature at 22% RH, (c) dynamic resistance curve of the sensor to 22% RH at 250 °C, and (d) dynamic response curves of the sensor to different concentrations of humidity (RH) at 250 °C.

Thus, the sensor’s dynamic resistance was measured at 250 °C under UV irradiation for different humidity concentrations (Figure 13d). The low detection limit was observed at 2.2% RH, at which point the resistance change was more than 1.5. The humidity concentration was swept to 98% RH, within which range the obtained curves showed reproducible behavior. At the highest concentration (98% RH), the response reached a value of 5, demonstrating the sensor’s maximum sensing capability. A strong linear response was observed over the RH detection range of 2.2%–84%, enabling reliable approximation of trace concentrations (Figure 14a). At the highest concentration, a clear deviation from the linear behavior was observed. This behavior is attributed to the complete occupation of available active sites on the sensor surface under supersaturated humidity conditions, thereby inhibiting further increases in response due to the saturation of water molecule adsorption.

**Figure 14:**
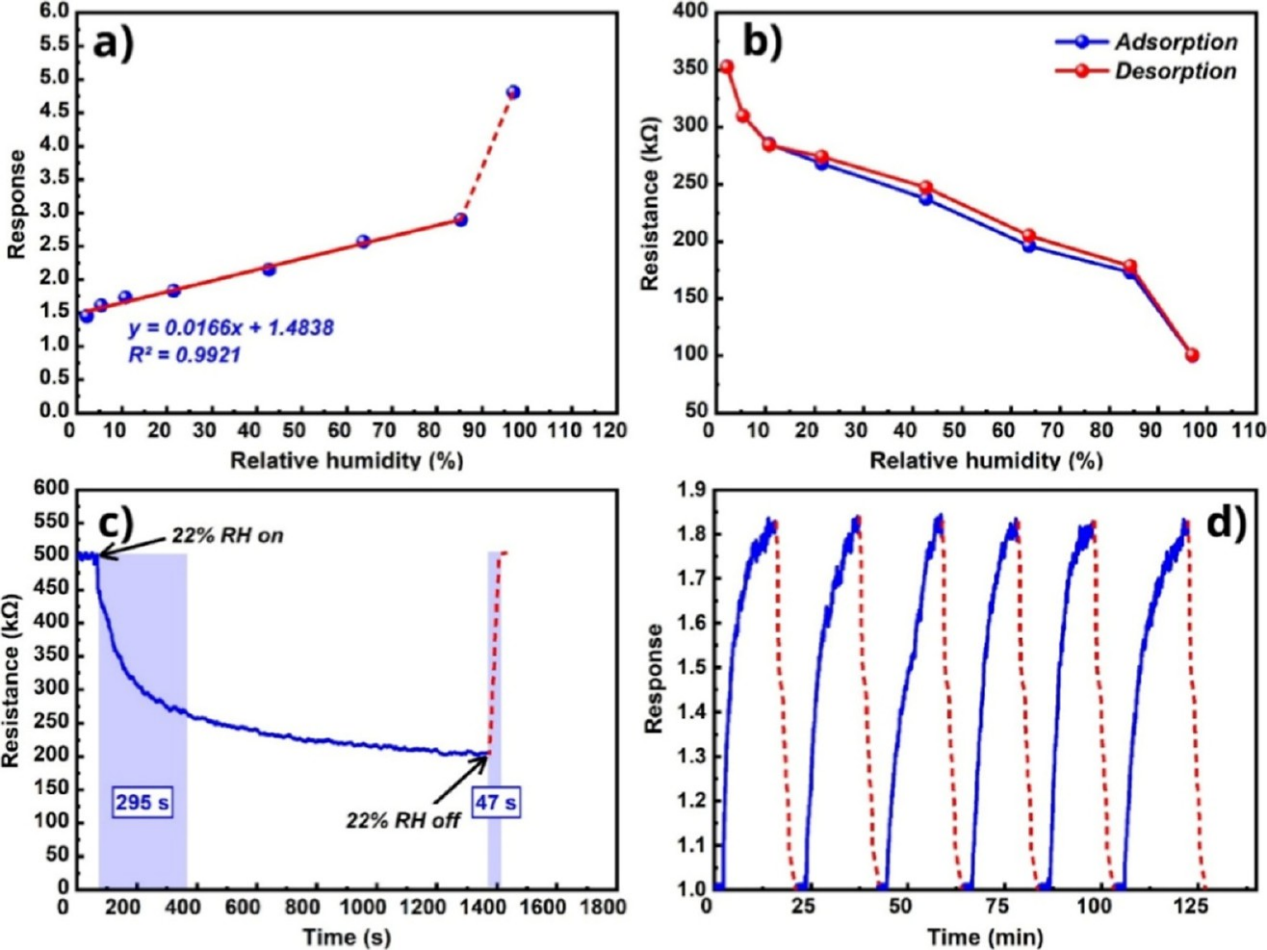
(a) Sensor response vs RH at 250 °C, (b) hysteresis curves of the sensor, (c) real-time response curve of the sensor at 22% RH, representing the response and recovery times at 250 °C, and (d) repeatability tests of the sensor to 22% RH at 250 °C.

Hysteresis is commonly observed in humidity sensors, indicating a mismatch between the adsorption and desorption trajectories.^57^ During the adsorption phase, water molecules readily undergo physisorption or chemisorption on the sensitive surface, whereas during desorption, their detachment from the surface is comparatively hindered.^58^ This means that the sensor’s resistance changes have difficulty tracking changes in the ambient humidity level, leading to humidity hysteresis. For the Fe_2_O_3_:ZnO/MWCNTs material, the largest resistance deviation was observed at 42.8% RH, and the hysteresis value did not exceed 4% (Figure 14b).

The real-time curves of the sensor resistance reflected the response and recovery times, which were 295 and 47 s, respectively (Figure 14c). The long response time was mainly due to the slowing down of diffusion processes, when the diffusion of water molecules to the surface of the sensitive layer and into its internal parts was slowed down due to the difficulties of their mobility in the bound state.^59^ In contrast, the recovery time was significantly reduced by the application of additional pulsed thermal heating. The increased thermal energy induced the rapid desorption of chemically bound water molecules, thereby restoring the sensor’s resistance to its baseline value (Figure 13c).

The repeatability of the sensor response under the same physical conditions was also checked multiple times. Dynamic response curves of the sensor were recorded in 6 identical measurements toward 22% humidity at 250 °C (Figure 14d). The near-coincidence of the sensor’s dynamic response curves demonstrated its high reproducibility and repeatability.

In resistive sensors, water vapor is generally regarded as an interfering species in target gas detection due to its pronounced cross-sensitivity.^60^ In the case presented here, where water vapor serves as the target analyte, no specific selectivity challenges were encountered. It is important here that the Fe_2_O_3_:ZnO/MWCNTs sensor showed extremely poor sensitivity to any other gas except humidity (Figure 15a). Thus, the sensor response to acetone (*n* = 800 ppm), ethanol (*n* = 700 ppm), propylene glycol (*n* = 600 ppm), carbon dioxide (*n* = 500 ppm), and methane (*n* = 500 ppm) was measured, with response values of 1; 1.1; 1.3; 1, and 1, respectively.

**Figure 15:**
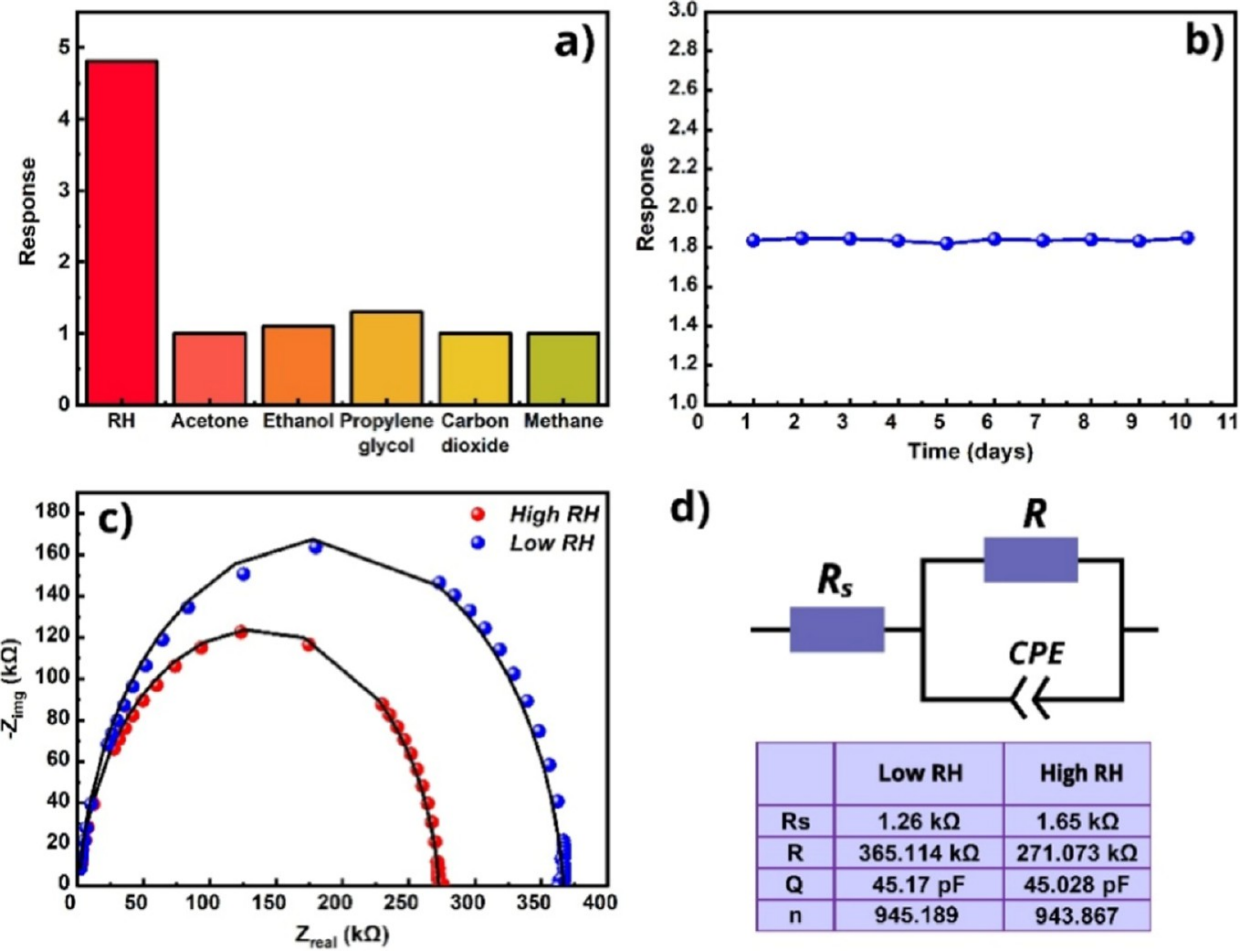
(a) Selectivity of the sensor for humidity at 250 °C, (b) long-term stability of the sensor for 22% RH at 250 °C, (c) sensor Nyquist curves in air and at 22% RH (b), and (d) the proposed equivalent electrical circuit of the sensor.

As is well-known, the stability of a resistive gas sensor refers to its ability to maintain a consistent and reproducible gas response over time under constant gas concentration and controlled operating conditions.^61^ For long-term and stable sensor operation, it is important to evaluate the response drift over time. Sometimes, the time factor causes deviations in the most important sensor parameters from their original values, which can be due to various factors. In this case, the most targeted key parameter is the gas response, which can monotonically decrease when the sensor is used repeatedly over days and weeks.^62^ This may be due to changes in the oxidation state of the metal oxide surface, adsorption of toxic molecules on the surface, agglomeration processes of nanoparticles within the film, etc.^63^ The sensor exhibited high temporal stability, with measurements conducted over 10 days showing negligible response drift at 22% RH and an operating temperature of 250 °C (Figure 15b).

The impedance measurements were also considered for Fe_2_O_3_:ZnO/MWCNTs material, and the parameter fitting was made with a 100 mV sinusoidal amplitude signal in a frequency range of 1 Hz–1 MHz. The sensor exhibited pronounced Nyquist semicircles in ambient conditions and a significant divergence of the curves in the presence of increased humidity (Figure 15c). The shift of the semicircle to the low resistance range in the presence of higher humidity was presented as an impedance response. An equivalent electrical circuit was also proposed for the sensitive film consisting of *R*_*s*_ (serial resistance), *R* (resistance), and CPE (constant phase element) (Figure 15d). Thus, under both ambient and elevated humidity conditions, the obtained Nyquist plots confirmed the presence of a capacitive component. However, the best-fitting circuit for these dependencies was the Rs-R||CPE structure.^64^ Under both environmental conditions, the CPE exponent (*n*) was close to unity, further confirming that this element exhibited predominantly capacitive behavior, while the parameter *Q* retained its characteristic value. The capacitance component expressed in this way was mainly related to the polycrystalline nature of the Fe_2_O_3_:ZnO/MWCNTs material, as well as the unique structure of the substrate.^65^ Regardless of these factors, the *R* element underwent a major change in humidity conditions, while the other elements of the equivalent electrical circuit almost did not change their values. The almost constant CPE values proved that water vapor did not penetrate the inner parts of the film, and surface processes predominated here.

The gas-sensing characteristics of the Fe_2_O_3_:ZnO/MWCNTs sensor were objectively compared with data from contemporary articles in the literature devoted to humidity detection (Table 2). Despite the high operating temperature, the sensor was characterized by its high response and a relatively low detection limit.

**Table 2:** Sensing Results of the Recently Published Humidity Sensors Compared to Those of Our Sensor

sensing materials	operating temperature [°C]	humidity range [%RH]	response	ref.
MWCNTs	23	90	55%	^66^
multilayer graphene	25	15–80	10%–17%	^67^
daily carbon ink	20	95	56%	^68^
MWCNT thin film	20	11	28%	^69^
chemically reduced graphene oxide (rGO)	25	80	1.78	^70^
PDDA/rGO	25	11–97	8.69%–37.43%	^71^
2-[2-(2-methoxyethoxy)ethoxy]ethylamine	80	97	31%	^72^
MnO_2_-coated CNT yarn	25	90	65%	^73^
rGO/MoS_2_ hybrid composite	25	10	9.03%	^74^
GO modified PEDOT: PSS	50	97	4.97%	^75^
Fe_2_O_3_:ZnO/MWCNTs	250	2.2	1.5	This work
Fe_2_O_3_:ZnO/MWCNTs	250	98	5	This work

### Humidity Sensing Mechanism

As a chemiresistive sensor, the gas-sensing mechanism is governed by the adsorption–desorption processes and surface chemical reactions involving water molecules on the sensing material.^76^ Here, the main mechanism is explained by Grotthuss principles, which establish proton conductivity on the surface of the sensitive material.^77^ Upon exposure to water vapor, they are transformed into the hydroxyl ions (OH^–^) and protons (H^+^) along the sensitive surface (eq 1). Hydroxyl ions can also be formed as a result of the interaction of oxygen ions (O^2–^ and O_2_^–^), electrons, and protons (eqs 2 and 3).^78^ The formation of hydroxyl ions and the increase in their concentration lead to the formation of a chemisorption layer. On the other hand, as the concentration of water molecules increases, the rate of their adsorption to the active surface increases, leading to the formation of a physical adsorption layer. In this case, protons combine with newly adsorbed water molecules to form H_3_O^+^ species that strongly support the electron transportation in the form of proton hopping (eq 4).^79^ This significantly contributes to the decrease in resistance, resulting in the expression of the response in the presence of humidity (Figure 16).

1 $$H_{2}O_{\left(aq.\right)}\to H_{\left(ads.\right)}^{+}+{OH}_{\left(ads.\right)}^{-}$$

2 $$H_{\left(ads.\right)}^{+}+O_{\left(ads.\right)}^{2-}\to {OH}_{\left(ads.\right)}^{-}$$

3 $$H_{2}O_{\left(aq.\right)}+1/2{O_{2}}_{\left(ads.\right)}^{-}+3/2e^{-}\to {2OH}_{\left(ads.\right)}^{-}$$

4 $$H_{2}O_{\left(ads.\right)}+H_{\left(ads.\right)}^{+}\to H_{3}O_{\left(ads.\right)}^{+}$$

**Figure 16:**
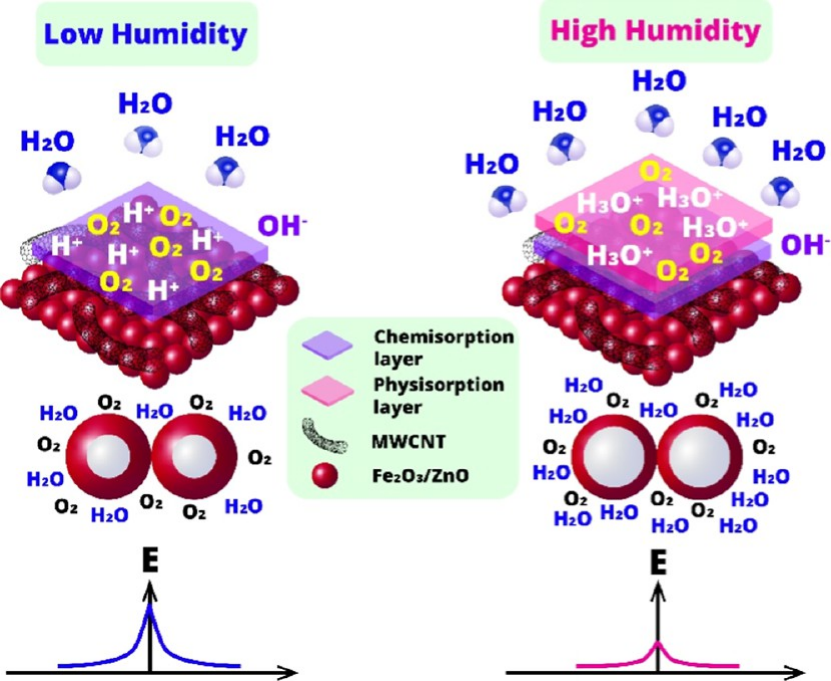
Humidity sensing mechanism for the Fe_2_O_3_:ZnO/MWCNTs material.

It is believed that MWCNTs contain various hydrophilic functional groups on their surfaces, which, in turn, create abundant adsorption sites that readily adsorb water molecules.^80^ Thus, by dramatically increasing the effective surface area of the composite material, nanotubes provide unique adsorption sites capable of absorbing large numbers of water molecules. This in itself leads to an increase in the humidity response, creating favorable conditions for the physisorption and chemisorption processes described above.

## Conclusions

This work presents the fabrication steps, characteristics, and humidity-sensing behavior of the Fe_2_O_3_:ZnO/MWCNTs nanocomposite material. The Fe_2_O_3_:ZnO target obtained by the simple solid-phase reaction method from 100 to 120 nm grain-sized nanopowder yielded a magnetron sputtered homogeneous film consisting of grains with an average size of 25–28 nm and a thickness of 36 nm. The TEM measurements of the Fe_2_O_3_:ZnO/MWCNTs material were carried out, attributed to interplanar distances of 0.5 and 0.25 nm for Fe_2_O_3_ and ZnO, respectively. The individual elements present in the material were confirmed by XPS and EDX studies, presenting their relative amounts (Fe - 59.85 wt %, O - 25.55 wt %, Zn – 12.28 wt %, and C - 2.32 wt %). The sensor’s most effective responsivity was recorded at 250 °C with combined UV irradiation as an alternative to enhance sensitivity toward humidity. Sensor recovery was supported by additional pulsed heating, enabling the sensor to quickly and completely reach baseline resistance levels. Superior humidity-sensing properties of the sensor presented a high response (*S* = 5, *n* = 98% RH), a wide detection range (2.2%–98% RH), a pronounced linearity in a range of 2.2%–84%, a low humidity hysteresis (4%), relatively short response and recovery times (295 and 47 s, respectively), a high reproducibility/repeatability, and an outstanding selectivity and temporal stability. An equivalent electrical circuit for the Fe_2_O_3_:ZnO/MWCNTs material was proposed, consisting of *R*_s_, *R*, and CPE elements, and a humidity sensing mechanism was discussed. To sum up, this paper presented a new outlook on the fabrication of high-performance humidity sensors and demonstrated significant potential across a wide range of applications.
